# Effect of Short Hydration on Cisplatin-Induced Nephrotoxicity in Cancer Patients: A Retrospective Study

**Published:** 2017-10-01

**Authors:** Farzaneh Ashrafi, Zeinab Ebrahimi, Mehdi Nematbakhsh

**Affiliations:** 1 Hematology and Medical Oncology Division, Internal Medicine Department, Isfahan University of Medical Sciences, Isfahan, Iran; 2 Acquired Immunodeficiency Research Center, Isfahan University of Medical Sciences, Isfahan, Iran; 3 School of Medicine, Isfahan University of Medical Sciences, Isfahan, Iran; 4 Department of Physiology, School of Medicine, Isfahan University of Medical Sciences, Isfahan, Iran; 5 Water and Electrolytes Research Center, Isfahan University of Medical Sciences, Isfahan, Iran

**Keywords:** Cisplatin, Nephrotoxicity, Hydration, Magnesium, Potassium

## Abstract

**Background: **The aim of this study was to evaluate the protective role of short hydration against nephrotoxicity induced by cisplatin (CDDP).

**Materials and Methods: **Twenty-two patients (13 men and 9 women) under CDDP therapy were enrolled in this retrospective study between 2009 and 2014. The CDDP was given in 500 ml of isotonic solution, and before and after CDDP administration, the patients received 10mEq potassium chloride15% and 1gr magnesium sulfate in 1000 ml isotonic saline. Renal parameters were evaluated on the first day of each cycle of CDDP therapy.

**Results: **Median cumulative CDDP dose was 465 mg/m^2^. Based on renal parameters, the prevalence of CDDP-induced nephrotoxicity (CIN) was 22.7%, while no hypokalemia and hypomagnesemia were observed.

**Conclusion: **Short hydration accompanied with potassium chloride and magnesium sulfate may decrease the risk of CIN.

## Introduction

 Cis*-*diamminedichloroplatinum(II)(Cisplatin, CDDP) is a chemotherapeutic drug used for treating broad spectrum of malignancies such as testis, ovary, bladder, head and neck, esophagus, breast, stomach and prostate, small and non-small cell lung cancer , Hodgkin^,^s and non-Hodgkin^,^s lymphomas, neuroblastoma, sarcoma, multiple myeloma , melanoma and mesothelioma ^1^^-^^5^ . However, CDDP therapy is limited by tumor cell resistance and severe side effects in normal tissues such as nephrotoxicity, neurotoxicity, ototoxicity, emetogenicity, myelosuppresion and immunosuppression ^1^^,^^2^^,^^5^^-^^7^ . Its main dose-limiting adverse effect is nephrotoxicity 2^,^^4^^-^^9^ . It is reported that nephrotoxicity is seen in approximately one-third of patients treated with a single dose (50mg/m^2^) of CDDP^   10 ^. In addition, about 20-40% of patients receiving high-dose of CDDP have severe renal dysfunction^2^^,^^4^^,^^8^^,^^11^^-^^13^. Usually, the dose and frequency of CDDP therapy, older age, female gender, current smoking, hypoalbuminemia and pre-existing renal dysfunction promote the CDDP-induced nephrotoxicity in patients  ^4^^,^^12^ . CIN could be recognized by decreased glomerular filtration rate (GFR), higher serum creatinine(sCr) and reduced serum magnesium and potassium levels ^2^^,^^5^ . Previous research indicated that sufficient hydration before and after administration of CDDP can reduce the induced nephrotoxicity^   8 ^. It is possible to reduce CIN but different applied strategies such as dose fractionation, screening for renal abnormalities, slower infusion rate, forced diuresis with diuretics and hydration could not completely abolished this side effect ^14^^,^^15^ . Although there are various hydrating protocols for CDDP, some components such as hydration volume and duration still remain controversial. Furthermore, the optimal intravenous solution and standard regimen for hydration are not completely clear^   14 ^. Accordingly, the present study was designed to evaluate the protective role of short hydration method using the isotonic saline fluid, magnesium sulfate (MgSo4) and potassium chloride (KCL) against CIN. 

## MATERIALS AND METHODS

**Patient selection:**

This retrospective study included 22 patients diagnosed with malignant tumors in Al-Zahra Hospital, Isfahan University of Medical Sciences from 2009 to 2014. All patients underwent chemotherapy consisted of CDDP. Patients were identified with cancer of lung, head and neck, esophagus, testis, ovarian, bladder, as well as Hodgkin’s lymphoma and refractory leukemia. 

The patients with the following characteristics were included to this study: histologically confirmed malignant tumor, candidate of chemotherapy with CDDP, age between 15 and 74 years, an Eastern Cooperative Oncology Group (ECOG) performance status of 0 or 1^   16 ^ and GFR more than 50 ml/min per 1.73 m^2^. Exclusion criteria were GFR less than 50 ml/min or use of nephrotoxic agents such as non-steroidal anti-inflammatory drugs and amino glycoside.

**CDDP administration protocol**:

Dose and the protocol of CDDP administration were modulated according to the drug therapeutic plan (alone or in association with other chemotherapeutic agents), depending on the tumor type and the condition of the patients. The mean quantity of CDDP administered was 50 – 100 mg/m^2^.

The CDDP was administered once every 21 days. All patients received 1000 mL isotonic saline plus 10 mEq KCl and 1 g MgSO4 during 2 hours before and after administration of CDDP. Its dose was calculated according to the body surface area and then administered as a 2-hour intravenous infusion in 500 mL of normal saline. 

**Measurement of renal parameters**

Complete blood cell and differential count were performed and routine chemistry determination was evaluated on the first day in every cycle of CDDP therapy. Nephrotoxicity was defined as an increase of 0.5 mg/dl or more above baseline sCr level during or immediately after CDDP infusion^   17 ^. Treatment-related toxicity was graded according to the National Institute Common Toxicity Criteria Version 4^   18 ^. The creatinine clearance was calculated with Cockcroft and Gault’s formula^   19 ^. Performance status was determined according to the Eastern Cooperative Oncology Group (ECOG) performance status scale^   17 ^.

Parameters for withholding CDDP were WBC< 3.0* 10^9^/l, neutrophil count <1.5*10^9^/l, platelet count < 100* 10^9^/l, sCr level > 1.4 mg/dl and ECOG performance status >2^   16 ^.

The definition of treatment response was evaluated according to the Response Evaluation Criteria In Solid Tumors (RECIST) criteria, version 1.1^   20 ^. Complete response (CR) was defined as the disappearance of all clinical disease evidence. Partial response (PR) was defined as a reduction in the sum of tumor measurements by at least 30%. Progressive disease (PD) was defined as a greater than 20% increase in the size of lesion or the appearance of any new lesions^   20 ^**. **If no response occurs during a period of 8 weeks, other modalities of treatment should be initiated.

**Statistical analysis:**

Data were analyzed using Statistical Package for the Social Sciences (SPSS) version 16.0. Paired T-test was used to determine the difference between sCr before and after chemotherapy, and the Wilcoxon Test was used for the values that were not normally distributed. The p-values ≤ 0.05 were considered as significant.

## Results

**Patient characteristics**

The patient’s demographic data and chemotherapy regimens administered are demonstrated in Tables 1 and 2, respectively. The median age of the patients was 44 (17-71) years including 13 (59.1%) men and 9 (40.9%) women. Meanwhile, 6 (27.3%) patients were stage III and 15 (68.2%) were stage IV. 

 The patients were subjected to receive several different CDDP combination drugs. However, in all patients, CDDP was administered at doses of 50-100 mg/m2. In this study, gemcitabine (Gem) was the most frequently used anticancer drug accompanied with CDDP (n=13, 59.1%). The mean (Min-Max) cumulative dose of CDDP administered was 480.91 (140-870) mg/m^2^. 

**Table 1:** Patients characteristics based on histology findings

Histology	Number of patients
**Lung** **Pancreas** **Ovary** **Germ cell Tumor** **Lymphoma** **Others** *	4 (18.2%) 4 (18.2%) 2 (9.1%) 2 (9.1%) 2 (9.1%) 8(36%)

* : Thymus, Stomach, Esophageal, Colon, Bladder, Uterus, Refractory Leukemia, Gall bladder

**Table 2:** The patients administered chemotherapy regimens

**Combination drug**	**Number of patients**
Gem – CDDP	14(63.6%)
BEP	2(9.1%)
Others*	6(27%)
Total number of cycles administered, median (range)	5 (1-6)
Three cycles or less	5 (22.7%)
Four cycles	4(22.7%)
Five cycles	2(9.1%)
Six cycles	10(45.5%)
Total dose of CDDP (mg/m^2^) Median ± SEM (Range)	465±42.63 (140-870)
Previous treatment with chemotherapy	Yes: 9 (40.9%) No: 13 (59.1%)

Gem-CDDP: Gemcitabine-Cisplatin, BEP: Cisplatin - Bleomycin – Etoposide,

* Agents combined with Cisplatin: Cytosar, Etoposide, Adriamycin, Cyclophosphamide, Taxol, Xeloda, Epirabicin, 5-Fluorouracil (5-FU)

**Evaluation of the renal parameters**

All patients who received CDDP had a normal sCr level (Table 3). Ten (45.5%) out of 22 patients completed the intended 6 cycles of chemotherapy. Only one patient experienced nephrotoxicity in the first cycle of CDDP therapy, and one patient experienced elevation in sCr after six cycles of chemotherapy.

During this study, CIN was observed in 5 patients (22.7%). Table 4 shows the characteristics of those who developed nephrotoxicity. Paired T-test analysis showed a significant difference in sCr level before the CDDP therapy and at the last visit (P=0.02). 

The levels of electrolytes in patients were evaluated on the first day of every cycle of CDDP and are demonstrated in Table 3. Hypokalemia and hypomagnesaemia were not observed in any patient.

**Table 3:** Renal parameters

**Laboratory parameters and** **reference values**	**Pre-CDDP** **(mean ** **±** ** SD)**	**Last visit** **(mean ** **±** ** SD)**
Creatinine (0.5 - 1.1 mg/dL)	0.89 ± 0.17	1.12 ± 0.48
Serum Mg concentration (1.2 - 2.6 mg/dL)	2.02 ± 0.27	2.03 ± 0.39
Serum K concentration (3.5 - 5.3 mEq/L)	4.15 ± 0.36	4.3 ± 0.50
GFR (ml/min/1.73 m2)	90.50 ± 17.89	80.13 ± 33.03

**Table 4:** Case characteristics of patients who developed nephrotoxicity

**No**	**Age ** **Sex**	**Diagnosis**	**Baseline sCr ** **(mg/dl) level**	**sCr level during ** **chemotherapy ** **(mg/dl)**	**Last visit SCr** **(mg/dl) level**	**Number of ** **chemo** **therapy ** **cycles**	**Treatment** **response**	**Interval ** **between ** **diagnosis and ** **last follow-up ** **(months)**
1	51/M	Pancreas	1.3	2.6	2.6	1	PD	2
2	30/M	Refractory ALL	1	0.8	1.5	2	PD	26
3	18/F	Lymphoma	0.7	0.8	1.6	2	PD	13
4	66/M	Gall Bladder	0.8	1	2	5	PD	8
5	64/M	Pancreas	0.8	1.6	1.2	6	PD	30

M: Male, F: Female, PD: Progressive disease

**Table 5:** Treatment responses

**Response**	**Patient Numbers**
CR	7 (31.8%)
PR+SD	6 (27.2%)
PD	8 (36.4)
CR then relapse	1 (4.5%)
Response rate	59%
OUTCOME	
Alive	16(72.7%)
Dead	6(27.3%)

CR: Complete response, PR: Partial response, SD: Stable disease, PD: Progressive disease

## Discussion

 The major side effect of CDDP is moderate to severe nephrotoxicity^   14 ^. Different strategies have been recommended to inhibit CIN. A number of animal studies suggested that various agents such as vitamin C and E, losartan, N-acetyl cysteine can be used as renoprotective against CIN ^3^^,^^21^^,^^22^ . Recently, published clinical guidelines have recommended hydration with normal saline before and after the administration of CDDP^   9 ^. There are many studies to evaluate the different hydration protocols ^1^^,^^9^^,^^15^^,^^16^^,^^23^^,^^24^ . However, some components such as appropriate amount and duration of hydration remain controversial. 

Ouchi et al., retrospectively compared patients who received outpatient chemotherapy containing CDDP (>60 mg/m^2^/ cycle) with the short hydration regimen (n=13) with those who received hospital chemotherapy with continuous hydration (n=17)^15^. In this study, the rate of acute kidney injury and increased sCr in the short hydration group were equal to or less than those in the continuous hydration group^   15 ^. Previous studies have recommend that 2 to 2.5 liters of hydration is required to allow safe administration of CDDP at a dose of >60mg/m^2 ^^15^^,^^16^ . In our study, CDDP was infused for 2 hours in 500 ml of isotonic solution. Two hours before and after the CDDP administration, the patients received isotonic saline 1000ml plus 10mEq KCL 15% and 1gr MgSo4. There was an increase in mean serum creatinine level and a decrease in the mean creatinine clearance in the study group after CDDP treatment. CIN was observed in 22.7% of patients.

Hypomagnesemia was observed in 50% of patients treated with CDDP-containing regimens. Hypomagnesemia causes direct cytotoxic damage of renal cells and enhances CIN^25^.

Several clinical trials examined the role of Mg supplementation in CIN. Yoshida et al. reported that the incidence of grade >2 sCr elevation in magnesium preloading group was significantly lower than non-magnesium preloading group during both the first cycle and all cycles^25^. The dosage of MgSo4 for supplementation therapy has varied in previous studies, ranging from 8 mEq to 2 gr ^24^^-^^26^ .

Kider et al. retrospectively evaluated potential risk factors for CIN as well as the potential impact of intravenous Mg supplementation on such toxicity in 401 cancer patients. Among the 52 patients who received Mg Supplementation, 6 (12%) developed hypomagnesaemia. A decrease in the serum magnesium concentration was observed in 20% of patients and was significantly associated with renal toxicity during the first course of cisplatin treatment^   26 ^. 

 Our treatment regimens included routine magnesium and potassium supplementation (intravenously) before and after CDDP infusion. Hypokalemia and Hypomagnesemia were not observed in any of the patients.

In our study population, CIN developed in one of the female patients. Few studies were published regarding sex difference in CIN. It is documented that there is a gender difference in CIN in the rat model and females have a lower risk for development and progression of chronic renal disease than males  ^27^^,^^28^ . Nematbakhsh et al. have demonstrated that the biochemical results revealed no specific sex-related differences, but the pathological data, kidney weight and weight loss were sex-dependent when a single dose of CDDP was administered in rats^   27 ^. In the cardiovascular system, protective role of estrogen before menopause is well known^   29 ^. However, estrogen did not attenuate the severity of CIN ^30^^, ^^31^.

Several limitations in this study need to be addressed in future research. First, it was a retrospective analysis of small numbers of patients (n=22). Second, CDDP was combined with other various cytotoxic agents in all patients. A prospective study with greater number of patients and a uniform protocol are needed to confirm the efficacy and safety of short CIN-induced nephrotoxicity. 

## CONCLUSION

 The results of this study have shown that short hydration accompanied with KCL and MgSo4 may decrease the risk of CIN. 
